# Fatal cardiac injury sustained from an air gun: Case report with review of the literature^☆^

**DOI:** 10.1016/j.ijscr.2020.04.039

**Published:** 2020-05-11

**Authors:** Timothy Guenther, Sarah Chen, Curtis Wozniak, David Leshikar

**Affiliations:** a Department of Surgery, University of California Davis, 2335 Stockton Blvd, 5th Floor, Sacramento, CA 95817, United States; b Department of Cardiothoracic Surgery, David Grant USAF Medical Center, 101 Bodin Cir, Travis Air Force Base, CA, United States

**Keywords:** Penetrating cardiac injury, Cardiac tamponade, Pellet gun injury, Air gun injury

## Abstract

**Introduction:**

Traditionally promoted as “toy guns,” air guns have long been used by children and lack many regulatory guidelines compared to conventional firearms. However these weapons possess serious lethal potential and have led to numerous injuries and deaths.

**Presentation of case:**

We describe a 21 year old man who sustained a penetrating cardiac wound from a pellet gun that led to cardiac tamponade and death. Post-mortem examination showed the pellet had penetrated the left ventricle and anterior esophagus with subsequent intraluminal migration into the stomach.

**Discussion:**

Review of the literature identified 39 other cases of penetrating cardiac injuries from air guns. Sternotomy was the most frequently used surgical approach and the right and left ventricles were the most commonly affected chambers. Bullet embolization was the most frequently reported complication. Including our case, five deaths related to penetrating cardiac injury from air guns were identified.

**Conclusions:**

This report highlights the seriousness of air guns and demonstrates a unique intra-thoracic injury.

## Highlights

• Air guns use compress air to cause bullet acceleration and can cause serious injury.
• Cardiac injuries from air guns are rare, but can lead to cardiac tamponade/death.
• Published cases of cardiac injuries from air guns most commonly occur in children.

## 1 Introduction

Air guns were first developed in the 16th century and have been used for hunting, war, and recreation [1]. These weapons use compressed air to cause bullet acceleration, in contrast to traditional firearms that use the ignition of explosive material for gas expansion and subsequent bullet acceleration [2]. Air guns operate by three potential mechanisms: a spring mechanism, pneumatic “hand-pump” mechanism, or supplemental CO_2_ delivery [3]. Air guns deliver a spherical shaped bullet (“BB”) or a cylindrical or modified cylindrical shaped bullet (“pellet”). Traditionally promoted as “toy guns,” air guns have long been used by children and lack many regulatory guidelines compared to conventional firearms [4,5]. The notion that air guns are safe or harmless is a common misnomer. Numerous reports have demonstrated the lethality of air guns [6]. We describe a 21 year old man who sustained a fatal penetrating cardiac injury from a pellet gun. This work has been reported in line with the SCARE criteria [7].

## 2 Presentation of case

A 21-year-old otherwise healthy man sustained a penetrating wound to the left chest from a pellet gun. The bullet was reported to have ricocheted off a wall and struck the patient’s anterior left chest. Further details related to the specifics of the shooting were unclear. A short time after the injury the patient was found unresponsive and 911 was called. On arrival, first responders found the patient asystolic and Advanced Cardiac Life Support (ACLS) was performed en-route to the hospital, with three rounds of epinephrine and two shocks delivered. A left needle thoracostomy was placed in the field without return of air or blood.

The patient arrived to our hospital’s trauma bay under CPR and was noted to have a small wound to his left anterior chest. The cardiac monitor showed an agonal rhythm and no cardiac motion was observed on ultrasound. Given the location of injury and suspicion for cardiac tamponade, a left anterolateral thoracotomy and right chest finger thoracostomy were performed. The pericardium was tense and a large amount of blood was evacuated after pericardiotomy. The heart was subjectively empty and cardiac massage continued while the patient was rapidly resuscitated with blood products. Upon inspection of the heart, there was a small hemostatic hole in the apex of the left ventricle. After adequate blood resuscitation and multiple rounds of CPR, he developed ventricular fibrillation for which he was shocked twice. His heart was full and still without a sustainable rhythm despite continued ACLS so further efforts were deemed futile and the patient was pronounced dead.

**Fig. 1:**
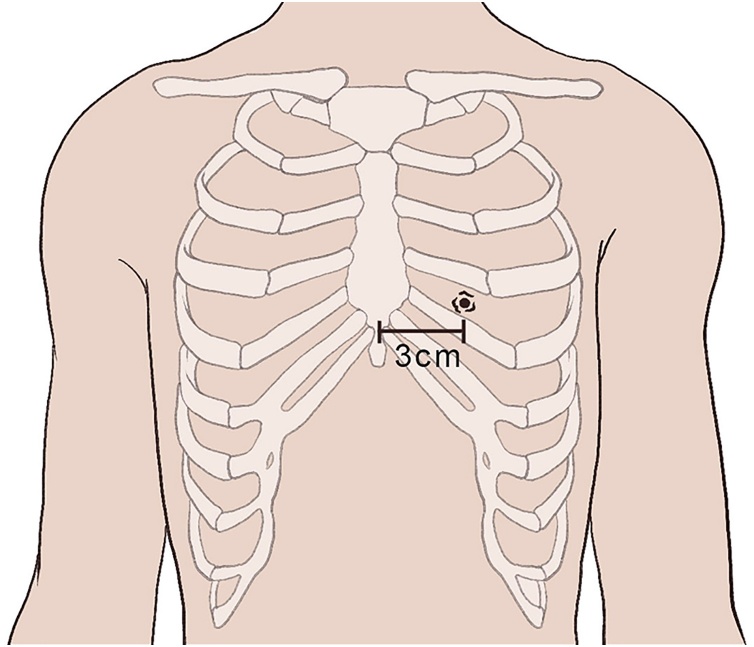
An illustration of the penetrating injury to the anterior chest wall is shown. The injury was located in the 6th intercostal space, 3 cm from the anterior midline.

**Fig. 2:**
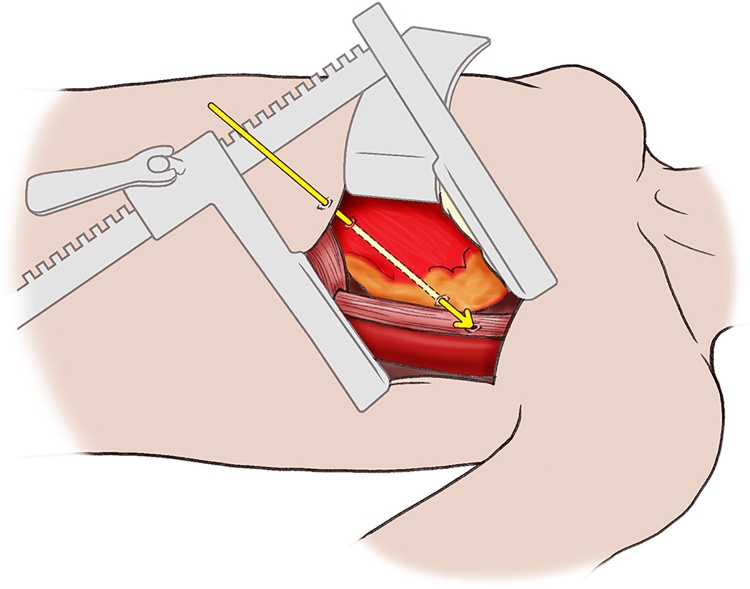
An illustration of the relationship between the penetrating injury to the heart and esophagus.

Post-mortem exam showed a small penetrating wound to the anterior 6th intercostal space 3 cm to the left of midline (Fig. 1). The bullet then coursed from the anterior apex of the left ventricle, through the posterior aspect of the base of the left ventricle, through the posterior pericardium, and through the anterior esophagus (Fig. 2). No defect was noted in the posterior esophagus and the bullet was recovered within the stomach. The presumptive cause of death was cardiac tamponade from the penetrating left ventricle wound which led to irreversible cardiovascular collapse.

## 3 Discussion

Gun violence is a serious public health crisis with an estimated 250,000 global deaths attributed to firearms in 2016 [8]. The amount of damage inflicted by a firearm is related to the kinetic energy the ballistic transfers to the target which is proportional to the mass and speed of the object (based on the equation E_k_ = ½ mv2 where E_k_ is kinetic energy, “m” is mass, and “v” is velocity) [9]. The mass of most BBs or pellets used in air guns is consistent between 0.12–0.43 g, however the velocity achieved can be quite variable depending of the mechanism used by the firearm and amount of air compression applied (for example how many pumps are applied to a pneumatic “hand-pump” air gun). The maximum average velocity achieved by air guns is between 290–940 feet/sec, compared to traditional firearms with an average velocity between 800–1200 feet/sec [10]. Prior studies have shown that a velocity of 191–331 feet/sec was needed to penetrate the skin and a velocity of 350 feet/sec was needed to penetrate bone [5,11]. Given the achievable velocities of air guns, the potential destructive ability of these devices is apparent. Numerous case reports/series have been published about injuries to the eye and brain from pellet guns, but there is a paucity of literature about cardiac injuries from air guns.

A review of the English language using Pubmed and Embase identified 40 cases of cardiac injuries sustained from air guns, including the case described (Supplement 1). Males were more commonly affected (90%) and the average age at presentation was 14 years (Table 1). Eighty percent of patients were ≤ 18 years of age. Forty eight percent of patients were hemodynamically unstable on presentation, based on description by the author of a systolic pressure < 90 mm Hg, heart rate >120, or altered mental status. A sternotomy was performed in 58% of cases; other approaches included thoracotomy and sub-xiphoid incisions. The right and left ventricles were equally the most affected chamber of the heart (both 33%). Bullet embolization was present in 25% of cases, requiring intervention in 6 cases. The case described in this report marks the fifth published case of an air gun causing a fatal cardiac injury globally and the second case in the US [3,9,12,13].

**Table 1:** Characteritics of Patients Who Sustained Cardiac Injury from Air Gun.

Patient Characteristic		Number of Patients	Percent of Patients
**Location of Author of Case Report**			
	North America	27	68%
	Europe	10	25%
	Asia	3	8%
**Gender**			
	Male	36	90%
	Female	4	10%
**Type of Air Gun Involved**			
	BB	10	25%
	Pellet	29	73%
	Not Specified “Air Gun”	1	3%
**Intervention Performed (not exclusive)**			
	Sternotomy	23	58%
	Use of Cardiopulmonary Bypass	7	18%
	Pericardial Window	6	15%
	Thoracotomy	5	13%
	Pericardiocentesis	4	10%
**Affected Cardiac Structure**			
	Left Ventricle	13	33%
	Right Ventricle	13	33%
	Interventricular Septum	11	28%
	Right Atrium	5	13%
	Interatrial Septum	4	10%
	Left Atrium	3	8%
	Named Coronary Vessel	3	8%
**Complication**			
	Bullet Embolization Requiring Intervention	6	15%
	Death	5	13%
	Bullet Embolization Not Requiring Intervention	4	10%
	Dysrhythmia	1	3%
	Aorto-cardiac Fistula	1	3%
	Post-op Pericardial Effusion Requiring Drainage	1	3%

Management of patients with penetrating thoracic injury from an air gun is similar to any other penetrating thoracic injury as defined by the principles put forth by Advanced Trauma Life Support (ATLS). A hemodynamically unstable patient requires prompt operative intervention to identify any potential source of correctable etiology, most commonly uncontrolled hemorrhage or cardiac tamponade in the setting of penetrating cardiac injuries [14]. In stable patients some advocate performing a pericardial window for any penetrating injury to the “box” (defined from the xiphoid inferior, bilateral nipples lateral and sternal notch superior) and if gross blood is encountered in the pericardium, a thorough inspection of the heart be performed via a median sternotomy [15]. However, some advocate for a more conservative approach to a positive pericardial window [16], but cases exist where expectant management in the setting of positive pericardial window after an air gun injury led to serious complications [17]. A chest x-ray and computerized tomography can additionally give more information in regards to the location of the bullet and bullet trajectory, but should only be used in a stable patient.

If operative intervention is necessary, a median sternotomy is considered the best exposure to evaluate the heart for an injury [18]. A left anterolateral thoracotomy can be performed rapidly in a patient who is decompensating and allows access to the pericardium to relieve potential cardiac tamponade. However, this exposure might make identification and/or repair of a cardiac injury challenging. Most cardiac injuries from air guns in our review occurred to the right and left ventricle, likely related to their relative anterior location in the pericardium, however injuries to every chamber of the heart have been identified. Intraoperative transesophageal echocardiogram can be used to evaluate the interatrial/ interventricular septum and valvular function, as the kinetic energy of an air gun ballistic is not always dissipated upon initial contact with the heart and additional internal cardiac injuries may be present [4].

If the kinetic energy of the bullet is dissipated on initial contact with the heart, the bullet can remain within the chamber of the heart and risks embolizing antegrade or retrograde. This phenomenon is not specific to air guns and has been well described with traditional firearms [19]. Although some bias likely exists from publishing unique cases, 25% of the cases in our review of air gun injuries to the heart were associated with embolization. Cases were found with antegrade and retrograde venous embolization as well as antegrade and retrograde arterial embolization. In the arterial system, the smaller mass of BBs/pellets can theoretically allow more distal embolization (to the popliteal or brachial artery) or embolization to the cerebral vasculature, compared to the larger mass of conventional firearm bullets that tend to embolize to the iliac, subclavian, or mesenteric arteries [19].

This case and the others described in our review again highlight the seriousness and potential lethality of air guns. An analysis from the United States Center for Disease Control showed that approximately 250,000 non-fatal injuries were sustained in the United States from air guns between 2001–2014 [20]. Unlike traditional firearms, air guns in the United States are not regulated at a federal level. Regulation instead falls upon each individual state, which can have considerable variability from no regulation to similar regulation as traditional firearms [5]. As echoed by the findings of our literature review, air gun injuries disproportionally affect younger patients, so more stringent regulation could limit unintended access of these devices which may decrease pediatric morbidity/mortality.

## 4 Conclusion

In conclusion, air guns should never be viewed as “toys” and should always be regarded in the same realm as conventional firearms given their potential for injury and lethality, as demonstrated by the case presented here.

## Declaration of Competing Interest

The authors disclose no conflicts of interest. The views expressed are that of the authors and do not reflect those of the Department of Defense or the United States Air Force.

## Sources of funding

The authors disclose no funding sources were used in the preparation of this manuscript.

## Ethical approval

This case report contained de-identified information and did not require ethical approval from our institution. Consent was obtained from both the patient’s mother and father to publish this case report.

## Consent

The patient presented in this case is deceased. Consent was obtained from both the patient’s mother and father to publish this case report.

## Author contribution

Timothy Guenther (TG).

Sarah Chen (SC).

Curtis Wozniak (CW).

David Leshikar (DL).

Study concept or design (TG, CW, DL), data collection (TG, SC, CW, DL), data analysis or interpretation (TG, SC, CW, DL), writing the paper (TG, SC, CW, DL).

## Registration of research studies

1. Name of the registry: NA (this was a single case report).
2. Unique identifying number or registration ID: NA (this was a single case report).
3. Hyperlink to your specific registration (must be publicly accessible and will be checked): NA (this was a single case report).

## Guarantor

Timothy Guenther is the guarantor for this work.

## Provenance and peer review

Editorially reviewed, not externally peer-reviewed.

## References

[1] Harris W., Luterman A., Curreri P.W. (1983). BB and pellet guns—toys or deadly weapons?. J. Trauma.

[2] Nakamura D.S., McNamara J.J., Sanderson L., Harada R. (1983). Thoracic air gun injuries in children. Am. J. Surg..

[3] Bligh-Glover W.Z. (2012). One-in-a-million shot: a homicidal thoracic air rifle wound, a case report, and a review of the literature. Am. J. Forensic Med. Pathol..

[4] Jackson C.C., Munyikwa M., Bacha E.A., Statter M.B., Starr J.P. (2007). Cardiac BB gun injury with missile embolus to the lung. J. Trauma.

[5] Presnell S.K. (2001). Federal regulation of BB guns: aiming to protect our children. NCL Rev..

[6] Kuligod F.S., Jirli P.S., Kumar P. (2006). Air gun—a deadly toy?: a case report. Med. Sci. Law.

[7] Agha R.A., Fowler A.J., Saetta A., Barai I., Rajmohan S., Orgill D.P., for the SCARE Group (2018). The SCARE statement: consensus-based surgical case report guidelines. Int. J. Surg..

[8] Naghavi M. (2018). Global mortality from firearms, 1990-2016. JAMA.

[9] Bakovic M., Petrovecki V., Strinovic D., Mayer D. (2014). Shot through the heart-firepower and potential lethality of air weapons. J. Forensic Sci..

[10] Stefanopoulos P.K., Pinialidis D.E., Hadjigeorgiou G.F., Filippakis K.N. (2017). Wound ballistics 101: the mechanisms of soft tissue wounding by bullets. Eur. J. Trauma Emerg. Surg..

[11] DiMaio V.J., Copeland A., Besant-Matthews P., Fletcher L.A., Jones A. (1982). Minimal velocities necessary for perforation of skin by air gun pellets and bullets. J. Forensic Sci..

[12] Milroy C.M., Clark J.C., Carter N., Rutty G., Rooney N. (1998). Air weapon fatalities. J. Clin. Pathol..

[13] Ng’walali P.M., Ohtsu Y., Muraoka N., Tsunenari S. (2001). Unusual homicide by air gun with pellet embolisation. Forensic Sci. Int..

[14] Bastos R., Baisden C.E., Harker L., Calhoon J.H. (2008). Semin. Thorac. Cardiovasc. Surg..

[15] Jhunjhunwala R. (2017). Reassessing the cardiac box: a comprehensive evaluation of the relationship between thoracic gunshot wounds and cardiac injury. J. Trauma Acute Care Surg..

[16] Thompson E.C., Block E.F., Mancini M.C. (1996). Management of BB shot wounds to the heart. J. Trauma.

[17] Fernandez L.G. (1995). Thoracic BB injuries in pediatric patients. J. Trauma.

[18] Besir Y. (2015). Choice of incision in penetrating cardiac injuries: which one must we prefer: Thoracotomy or sternotomy?. Ulus. Travma Acil Cerrahi Derg. TJTES.

[19] Kuo A.H., Gregorat A.E., Restrepo C.S., Vinu-Nair S. (2019). Systematic review of civilian intravascular ballistic embolism reports during the last 30 years. J. Vasc. Surg..

[20] Nonfatal Injury Reports, 2001–2014, https://webapp.cdc.gov/sasweb/ncipc/nfirates2001.html.

