# Supplementary material for: Fatal cardiac injury sustained from an air gun: Case report with review of the literature
Source: Int J Surg Case Rep. 2020 May 11;70:133–6. doi: 10.1016/j.ijscr.2020.04.039 (PMC7229402; doi:10.1016/j.ijscr.2020.04.039)
Supplement: Supplementary file 1 [file mmc1.pdf]

Supplement 1 (not the manuscript)

Cases of Cardiac Injury from Air Gun[1-33]

1 Alejandro, K. V., Acosta, J. A. & Rodriguez, P. A. Air gun pellet cardiac injuries: case report and review of the literature. *The Journal of trauma* **54**, 1242-1244, doi:10.1097/01.ta.0000071290.41225.ab (2003).

2 Schowengerdt, C. G., Vasko, J. S., Craenen, J. M. & Teske, D. W. Air gun pellet injury of the heart with popliteal embolus. *The Annals of thoracic surgery* **40**, 393-395 (1985).

3 Babin-Ebell, J., Graf, B., Sievers, H. H. & Duebener, L. F. An air gun pellet retained in the heart: a case report. *The heart surgery forum* **11**, E127-128, doi:10.1532/hsf98.20071208 (2008).

4 Isik, O., Engin, C., Daylan, A. & Sahutoglu, C. Air gun pellet: cardiac penetration and peripheral embolization. *Ulusal travma ve acil cerrahi dergisi = Turkish journal of trauma & emergency surgery : TJTES* **22**, 301-303, doi:10.5505/tjtes.2015.76570 (2016).

5 Friedman, D., Hammond, J., Cardone, J. & Sutyak, J. The air gun: toy or weapon? *South Med J* **89**, 475-478, doi:10.1097/00007611-199605000-00004 (1996).

6 Morales, J. M., Patel, S. G., Monarrez, C., Saldehar, A. & Simpson, J. W. Air pellet embolization after penetrating cardiac injury. *The Journal of trauma* **49**, 774-775 (2000).

7 Waters, D., Broghammer, B. & Duff, R. Air pellet gun injury. *Iowa medicine: journal of the Iowa Medical Society* **85**, 331 (1995).

8 Wascher, R. A. & Gwinn, B. C., 2nd. Air rifle pellet injury to the heart with retrograde caval migration. *The Journal of trauma* **38**, 379-381 (1995).

9 Milroy, C. M., Clark, J. C., Carter, N., Rutty, G. & Rooney, N. Air weapon fatalities. *Journal of clinical pathology* **51**, 525-529, doi:10.1136/jcp.51.7.525 (1998).

10 Bond, S. J., Schnier, G. C. & Miller, F. B. Air-powered guns: too much firepower to be a toy. *The Journal of trauma* **41**, 674-678 (1996).

11 Neerken, A. J. & Clement, F. L. Air-rifle wound of the heart with embolization. *JAMA* **189**, 579-580 (1964).

12 Misseldine, S. & August, D. A. Anesthesia for a thoracic BB gun injury. *Paediatric anaesthesia* **20**, 566-573, doi:10.1111/j.1460-9592.2010.03306.x (2010).

13 Avsar, M. K., Demir, S., Onsel, I. O. & Poyrazoglu, H. H. Aorta-to-Left Atrial Fistula Caused by Air Gun Pellet Cardiac Injury. *Cardiology and therapy* **3**, 67-71, doi:10.1007/s40119-014-0026-7 (2014).

14 Hudson, A. J. & Wyatt, J. P. Cardiac air gun pellet injury. *Emergency medicine journal : EMJ* **18**, 519, doi:10.1136/emj.18.6.519 (2001).

15 Baruah, N., Talukdar, M., Choudhury, D. & Raibaruah, A. J. Cardiac air rifle pellet injury: a case report. *Indian Journal of Thoracic and Cardiovascular Surgery* **27**, 45-47 (2011).

16 Jackson, C. C., Munyikwa, M., Bacha, E. A., Statter, M. B. & Starr, J. P. Cardiac BB gun injury with missile embolus to the lung. *The Journal of trauma* **63**, E100-104, doi:10.1097/TA.0b013e3181469ea7 (2007).

17 Mingorance, M. A., Casado, F. C., Mendias, J. V., López-Tello, C. S. & de la Cruz, J. P. Cardiac injury from an air gun pellet: A case report. *European journal of pediatric surgery* **9**, 184-185 (1999).

18 Suchedina, A. A., Watson, D. C. & Alpert, B. S. Cardiac injury from an air gun pellet: a case report. *American Journal of Diseases of Children* **147**, 262-263 (1993).

19 Barnes, F. & Helson, R. A death from an air gun. *Journal of Forensic Science* **21**, 653-658 (1976).
20 Willemsen, P., Kuo, J. & Azzu, A. Dysrhythymia from an intrapericardial air gun pellet: a case report. *European journal of cardio-thoracic surgery : official journal of the European Association for Cardio-thoracic Surgery* **10**, 461-462, doi:10.1016/s1010-7940(96)80116-3 (1996).
21 Lamb, R. K., Pawade, A. & Prior, A. L. Intravascular missile: apparent retrograde course from the left ventricle. *Thorax* **43**, 499-500, doi:10.1136/thx.43.6.499 (1988).
22 DeCou, J. M., Abrams, R. S., Miller, R. S., Touloukian, R. J. & Gauderer, M. W. Life-threatening air rifle injuries to the heart in three boys. *Journal of pediatric surgery* **35**, 785-787, doi:10.1053/jpsu.2000.6079 (2000).
23 Robison, R. J., Brown, J. W., Caldwell, R., Stone, K. S. & King, H. Management of asymptomatic intracardiac missiles using echocardiography. *The Journal of trauma* **28**, 1402-1403 (1988).
24 Thompson, E. C., Block, E. F. & Mancini, M. C. Management of BB shot wounds to the heart. *The Journal of trauma* **40**, 168-170 (1996).
25 Klein, J. A., Nowak, J. E., Sutherell, J. S. & Wheeler, D. S. Nonsurgical management of cardiac missiles. *Pediatric emergency care* **26**, 36-38, doi:10.1097/PEC.0b013e3181c39a39 (2010).
26 Sanchez, E. *et al.* Observational management in a case of cardiac air gun pellet injury. *Injury Extra* **43**, 4-5 (2012).
27 Bligh-Glover, W. Z. One-in-a-million shot: a homicidal thoracic air rifle wound, a case report, and a review of the literature. *The American journal of forensic medicine and pathology* **33**, 98-101, doi:10.1097/PAF.0b013e318221b8a9 (2012).
28 Chew, J. D., Nicholson, G. T., Mettler, B. A. & Doyle, T. P. Percutaneous Removal of Intravascular Pellet Following Penetrating Cardiac Trauma. *Pediatric cardiology* **39**, 191-194, doi:10.1007/s00246-017-1689-3 (2018).
29 Bakovic, M., Petrovecki, V., Strinovic, D. & Mayer, D. Shot through the heart-firepower and potential lethality of air weapons. *Journal of forensic sciences* **59**, 1658-1661, doi:10.1111/1556-4029.12486 (2014).
30 Doetsch, N., Wolfhard, U., Mathers, M. J. & Zerkowski, H. R. Survival after heart and coronary-artery penetration by an air-rifle projectile. *The Thoracic and cardiovascular surgeon* **37**, 332-334, doi:10.1055/s-2007-1020348 (1989).
31 Nakamura, D. S., McNamara, J. J., Sanderson, L. & Harada, R. Thoracic air gun injuries in children. *The American Journal of Surgery* **146**, 39-42 (1983).
32 Dawson, J., Rodriguez, Y., Pham, S. M. & Ferreira, A. Traumatic transection of the left anterior descending artery caused by a projectile. *Journal of cardiology cases* **5**, e140-e142 (2012).
33 Ng'walali, P. M., Ohtsu, Y., Muraoka, N. & Tsunenari, S. Unusual homicide by air gun with pellet embolisation. *Forensic science international* **124**, 17-21 (2001).
